# Supplementary material for: Predictors of delayed initiation of breast milk and exclusive breastfeeding in Ethiopia: A multi-level mixed-effect analysis
Source: PLoS One. 2024 Apr 3;19(4):e0301042. doi: 10.1371/journal.pone.0301042 (PMC10990229; doi:10.1371/journal.pone.0301042)
Supplement: Attachment — Submitted filename: Response to Reviewers.docx [file pone.0301042.s001.docx]

**Author Response to Editor and Reviewers**

**Dear Editor and Reviewers,**

We would like to express our gratitude for your email dated 26 Feb 2024, which included the insightful comments from the editor and reviewers. We, the authors, sincerely appreciate the valuable and constructive review that has greatly contributed to the improvement of our paper titled **"Predictors of Delayed Initiation of Breast Milk and Exclusive Breastfeeding in Ethiopia: A Multi-level Mixed-effect Analysis".** We have thoroughly reviewed the editor's and reviewers' comments and have made the necessary revisions to the manuscript accordingly. Our responses to the editor and reviewers' comments are provided in a point-by-point manner using the Author's response to reviewer form. If you have any further concerns or suggestions, we are more than willing to address them.

Best regards,

Version 1: PONE-D-23-34507

Date: 2/27/2024

**Academic editor comments and respective author’s response**

**Editor comment 1:** Please ensure that your manuscript meets PLOS ONE's style requirements, including those for file naming. The PLOS ONE style templates can be found at [**https://journals.plos.org/plosone/s/file?id=wjVg/PLOSOne_formatting_sample_main_body.pdf**](https://journals.plos.org/plosone/s/file?id=wjVg/PLOSOne_formatting_sample_main_body.pdf) **and** [**https://journals.plos.org/plosone/s/file?id=ba62/PLOSOne_ formatting_ sample_ title_authors_affiliations.pdf**](https://journals.plos.org/plosone/s/file?id=ba62/PLOSOne_%20formatting_%20sample_%20title_authors_affiliations.pdf)

**Authors Response:** Thanks very much for this comment. The whole part of the manuscript has been documented as per the PLOS ONE style templates.

**Editor comment 2:** Please provide additional details regarding participant consent. In the ethics statement in the Methods and online submission information, please ensure that you have specified (1) whether consent was informed and (2) what type you obtained (for instance, written or verbal, and if verbal, how it was documented and witnessed). If your study included minors, state whether you obtained consent from parents or guardians. If the need for consent was waived by the ethics committee, please include this information.

**Authors Response:** Thanks very much for this comment. We used an aggregated publicly available secondary data taken from the Measure DHS program (<http://www.dhsprogram.com>), which does not include any personal identifiers that could be linked to the study participants **(*See page 9, under the sub-heading*** ***‘‘Ethical consideration’’***).

**Editor comment 3:** Note from Emily Chenette, Editor in Chief of PLOS ONE, and Iain Hrynaszkiewicz, Director of Open Research Solutions at PLOS: Did you know that depositing data in a repository is associated with up to a 25% citation advantage……

**Authors Response:** Thanks very much for this supportive comment. We updated the data availability statement as ‘‘We declared that all the data underlying the results presented in the study are publicly available from the Harvard Dataverse; <https://doi.org/10.7910/DVN/YISAFE>’’.

**Editor comment 4:** We note that Figure 1 in your submission contain map/satellite image which may be copyrighted. All PLOS content is published under the Creative Commons Attribution License (CC BY 4.0), which means that the manuscript, images, and Supporting Information files will be freely available online, and any third party is permitted to access, download, copy, distribute, and use these materials in any way, even commercially, with proper attribution……

**Authors Response:** Thanks very much for this insightful comment. We, the authors, assure that the map used in figure 1 are freely available at: <https://africaopendata.org/dataset/ethiopia-shapefiles>.

**Editor comment 5****:** Please review your reference list to ensure that it is complete and correct…..

**Authors Response:** Thanks very much for this constructive comment. We, the authors, reviewed, checked, and ensured that all the reference lists were complete and correct.

**Reviewer #1 comments and an author’s response**

Dear PLOS One team of editorials, thank you for giving me the chance to review the manuscript entitled " Predictors of Delayed Initiation of Breast Milk and Exclusive Breastfeeding in Ethiopia: A Multi-level Mixed-effect Analysis”

This study gives very important results regarding delayed initiation of breast milk and exclusive breastfeeding. However, in a few areas, here are my comments.

**Comment #1:** While reading the abstract …Which software you used for data analysis is not clear ....it must be included in the abstract…. The abstract is many-worded try to make it short, catchier, and reader-friendly... conclusion does not match with the objective.

**Authors Response:** Thanks very much, dear reviewer, for these insightful comments. We accept the comments and corrections have been made accordingly (***See pages 2 to 3 under the heading ‘‘Abstract’’***).

**Comment #2:** WHO recommends exclusive breastfeeding for the first 6 months of life, but in your study, you include ages 0-5 ...why??

**Authors Response:** Thanks very much, dear reviewer, for this critical comment. The WHO define exclusive breast feeding as the infant received only breast milk without any additional food or drink, including water, ***for the first 6 months of life (0-5 months),*** except for vitamins, mineral supplements, or medicines ***(WHO, 2023)***.

*WHO: Exclusive breastfeeding for optimal growth, development and health of infants; Updated on 9 August 2023. Available at; https://*[*www.who.int/tools/elena/interventions/exclusive-breastfeeding#:~:text=Exclusive%20breastfeeding%20means%20that%20the,of%20vitamins%2C%20minerals%20or%20medicines*](http://www.who.int/tools/elena/interventions/exclusive-breastfeeding#:~:text=Exclusive%20breastfeeding%20means%20that%20the,of%20vitamins%2C%20minerals%20or%20medicines)*. 2023.*

**Comment #3:** Keywords are not matched with the title

**Authors Response:** Thanks very much, dear reviewer, for this insightful comment. We accept the comment and correction has been made accordingly (***See page 3, line 9***).

**Comment #4:** In the background major consequences or effects of delayed initiation of feeding or not doing exclusive breastfeeding are not discussed.

**Authors Response:** Thanks very much for this constructive comment. The major consequences or effects of delayed initiation of feeding or not doing exclusive breastfeeding are discussed under the introduction section, paragraph two *(****See page 3, lines 15–22****).*

**Comment #5:** There are dissimilarities in study objectives: in abstract and introduction

**Authors Response:** Thank you very much for your insightful comment. We greatly appreciate it. The comment has been accepted, and revisions have been made to ensure that the objectives in the abstract section are aligned with the introduction ***(See page 2, lines 7-8, and page 5, lines 9-11).***

**Comment #6:** Revisit the numbers, totals, and the statistics in general.

**Authors Response:** Thanks very much, dear reviewer, for this insightful suggestion. We, the authors, assured that all the numbers and percentages have been checked and all are correct.

**Comment #7:** Insufficient citation, particularly in discussion for safe interpretation.

**Authors Response:** Thanks very much, dear reviewer, for this insightful comment. We, the authors, accept the comment and revised the citation in the discussion section accordingly.

**Comment #8:** It is not mandatory to report the strengths of your study.

**Authors Response:** Thank you very much, dear reviewer, for your insightful suggestion. We, the authors, genuinely appreciate your concern. However, we are keen on providing a comprehensive explanation of both the strengths and limitations of the study and addressed under the section **‘‘strength and limitations of the study’’.**

**Comment #9:** What are the measures taken to control the confounders?

**Comment #10:** Use correct tense, grammar, sentence, spelling, paraphrase, consistency. check it.

**Authors Response:** Thanks very much, dear reviewer, for this insightful suggestion. We, the authors, assured that all things included in the document have been checked and correct.

**Reviewer #2 comments and authors’ response**

**Comment #1:** Sampling was done about 5 years ago. Does this data look old? Considering the importance of publishing DHS data, how do the authors justify the reason for this delay?

**Authors Response:** Thank you, dear reviewer, for your questions. We, the authors, confirm that the data used in our study was obtained from the ***most recent*** Ethiopia Mini Demographic and Health Survey 2019. This survey collected information from respondents within the five years prior to the survey period. The survey took place from March 21^st^, 2019 to June 28^th^, 2019 ***(EPHI, 2019).***

*Ethiopia Public Health Institute, ICF: Ethiopia mini demographic and health survey 2019: key indicators. Rockville, Maryland, USA: EPHI and ICF 2019.*

**Comment #2:** One of the cases that seems to be necessary to be considered in the criteria for selecting people is the cases where it is not possible to start early feeding with breast milk after giving birth due to maternal and infant problems, which is a completely scientific and logical separation. When the results of the delay in the initiation of breastfeeding for this high-risk mother or infant are combined with the delay in the onset of breastfeeding (newborns or mothers who did not have any problems after delivery), it leads to a higher indication of the delay in the initiation of breastfeeding. How did the researchers deal with this problem?

**Authors Response:** Thank you, dear reviewer, for your insightful comment and question. We, the authors, appreciate your concern. As we utilized secondary data, the variables regarding maternal and infant problems were not specifically documented, leading to our inability to classify which women or newborns experienced problems after delivery. We acknowledge this as a limitation of our study and have included it in the "**Strengths and Limitations**" section ***(See page 23, line 7).***
